# From Rags to Riches: Assessing poverty and vulnerability in urban Nepal

**DOI:** 10.1371/journal.pone.0226646

**Published:** 2020-02-05

**Authors:** Tim Ensor, Radheshyam Bhattarai, Shraddha Manandhar, Ak Narayan Poudel, Rajeev Dhungel, Sushil Baral, Joseph P. Hicks, Dana Thomson, Helen Elsey

**Affiliations:** 1 Nuffield Centre for International Health and Development, University of Leeds, Leeds, England, United Kingdom; 2 HERD INTERNATIONAL, Kathmandu, Nepal; 3 Helen Keller International, Lalitpur, Nepal; 4 World Vision International Nepal, Kathmandu, Nepal; 5 Social Statistics, University of Southampton, Southampton, England, United Kingdom; Leibniz Institute for Prevention Research and Epidemiology BIPS, GERMANY

## Abstract

Urbanisation brings with it rapid socio-economic change with volatile livelihoods and unstable ownership of assets. Yet, current measures of wealth are based predominantly on static livelihoods found in rural areas. We sought to assess the extent to which seven common measures of wealth appropriately capture vulnerability to poverty in urban areas. We then sought to develop a measure that captures the characteristics of one urban area in Nepal. We collected and analysed data from 1,180 households collected during a survey conducted between November 2017 and January 2018 and designed to be representative of the Kathmandu valley. A separate survey of a sub set of households was conducted using participatory qualitative methods in slum and non-slum neighbourhoods. A series of currently used indices of deprivation were calculated from questionnaire data. We used bivariate statistical methods to examine the association between each index and identify characteristics of poor and non-poor. Qualitative data was used to identify characteristics of poverty from the perspective of urban poor communities which were used to construct an Urban Poverty Index that combined asset and consumption focused context specific measures of poverty that could be proxied by easily measured indicators as assessed through multivariate modelling. We found a strong but not perfect association between each measure of poverty. There was disagreement when comparing the consumption and deprivation index on the classification of 19% of the sample. Choice of short-term monetary and longer-term capital approaches accounted for much of the difference. Those who reported migrating due to economic necessity were most likely to be categorised as poor. A combined index was developed to capture these dimension of poverty and understand urban vulnerability. A second version of the index was constructed that can be computed using a smaller range of variables to identify those in poverty. Current measures may hide important aspects of urban poverty. Those who migrate out of economic necessity are particularly vulnerable. A composite index of socioeconomic status helps to capture the complex nature of economic vulnerability.

## Introduction

Rapid urbanisation is changing the nature of household wealth and poverty in low- and middle-income countries (LMICs). Compared to rural settings, urban households must typically spend more on housing, food, education and health-care and yet, despite notions of ‘urban advantage’, those in poor neighbourhoods and slums frequently have worse health outcomes than their rural counterparts [1]. Given the dynamic context of urban areas, shaped by rural to urban migration, the appropriateness of common measures of wealth used in cross-sectional and other epidemiological studies in LMICs has been questioned [2, 3]. Within LMIC contexts, the results of these cross-sectional studies are of particular importance as they are often the main source of reliable data on which to measure key health and social sector outcomes and to inform policy and plan the response. An appropriate measure of economic vulnerability is therefore crucial if resources and activities are to be targeted to the urban poor and ultimately reduce inequities.

Our objective was to develop a usable and sensitive measure of urban household poverty that can be used as the basis for understanding health variation across households and targeting services and other social programmes towards those in most need. We first focus on understanding how seven measures of poverty (income per capita, consumption per capita, asset index, progress out of poverty, index of deprivation, UN Habitat slum index, self-identification as poor) vary using different classification methods, their overlap and differences. We then examine the way in which urban communities understand differences between poor and rich households and individuals. This information is then used to produce a combined measure of acute and chronic poverty that can be estimated using easily available household indicators.

Assessments of absolute poverty levels frequently begin by examining household income. While this provides an intuitive measure of household resource availability, it also has several drawbacks. One is that assessing household income is often complex resulting in imprecise estimates [4]. Individuals often have multiple income generating activities some of which may only last a short period of time which require extensive and complex instruments to measure [5]. In the urban context, where many poor households are reliant on unpredictable daily-wage employment, this is a particular challenge. Individuals are often also reluctant to share details of their income. Where income is derived from questionable sources, such as ‘protection’ money paid by slum-dwellers to local gangs or ‘leaders’ to ensure their safe residence in the slum, urban dwellers are unlikely to share details. A commonly used alternative is to document household expenditure. This is often easier to do but is also problematic [5]. In urban contexts where households must buy many goods and food items, relying less on subsistence than in the rural area, and are able to buy these in multiple retail outlets all with different prices, recalling expenditure is particularly challenging.

Calculating resources at the household level says nothing about how resources are distributed within a household. Studies have demonstrated that there is often substantial intra-household inequity relating to the gender and age structure of the household itself [6, 7]. Within the urban context, household structures are changing with women as well as men now working for an income, potentially affecting power structures and control of resources. This is an important critique although one that applies to many of the household measures including those that focus on assets.

A further problem with consumption measures, highlighted by the capability theory of poverty attributed to Sen and others, is that there is variation in the way in which individuals or households convert levels of financial (or other) resources into capabilities and wellbeing [8]. Again, this critique can be applied to asset approaches while broader approaches may attempt to capture the achievement of capabilities through assessment of other characteristics such as education or health.

A third criticism of the consumption or income approach, less applicable to asset assessment, is that annual resources can and often do exhibit substantial fluctuation [9], with the instability in employment in urban areas, this is particularly relevant in the context of urbanisation. Unless data are longitudinal, therefore, consumption and income provide only a transitory view of resource availability. More prosaically, expenditure or consumption itself requires an extensive instrument often based on a diary approach that can be time consuming to obtain. In urban areas, where poor households work long hours, this can present further challenges in response rates to household surveys.

An alternative to measuring current resources is to assess assets. Assets are attractive because they are often easier to measure and verify and as a result have become the standard method of assessing wealth in the Demographic and Health Survey (DHS) and similar surveys [10]. Assets may also be a better measure of longer term or chronic poverty since they represent past purchasing power as well as current resources. Conversely, however, assets may fail to capture recent or acute hardship preventing households meeting basic needs such as nutrition since assets are ‘slow moving and discrete’ [11]. While assets can be sold to provide immediate resources, this may take time and depends on a market for the asset in question. Although assets do not fully represent capabilities, in some cases they can provide a better proxy than money resources. In the case of water, for example, owning or having access to a standpipe provides a clearer indication of access to clean water than expenditure on the same item which, particularly if a household is forced to purchase bottled or packet water, may be inadequate to meet clean-water needs.

Other measures of deprivation broaden the asset perspective to capture measurable capabilities of individuals and households. Multidimensional approaches such as Bag and Seth’s deprivation index focus on indicators that assess household capabilities including water and sanitation availability, housing, respiratory risk, education attainment and access to information via mobile phones or equivalent, but do not include measures of current consumption or income [3]. These measures permit a focus on the results and impact of resource availability on access to key functions that determine household and individual welfare.

The measure of poverty chosen may depend in part on the objective of the programme for which targeting is required. Programmes targeted at ensuring families have adequate nutrition, for example, might concentrate on current assessment of resource availability as measured by consumption or income. Conversely, programmes aimed at redistributing resources to ensure improved longer term livelihoods may focus more on broader availability of assets and household capital. This possible dichotomy has limitations. Even if a programme is largely focused on providing a safety net against starvation, assets such adequate sanitation, cooking facilities and basic education remain important to enhance an individual’s capability to achieve improved welfare [3]. Similarly, long term livelihoods, partly captured by asset availability also require that citizens have adequate current income to meet daily subsistence needs. An adequate measure of poverty needs to reflect both acute needs and longer term chronic vulnerability.

A number of methods for identifying the poor are in operation in Nepal. Geographic targeting is common for some services. The Human Development Index was used to identify the poorest 25 districts for implementation of free maternal health care [12]. More recently, the country has begun to use a multidimensional index of poverty to assess living standards at a provincial and sub-provincial level in order to assist in targeting resources [13]. This assesses household physical (assets, housing etc.) and human (education, health) capital leading to an index of impoverishment. At a household level, poor household cards are distributed to three categories of households–very poor, medium and marginalised [14]. Assessment is based on a proxy means test (PMT) that draws on a combination of household specific indicators, such as property holding, and community indicators, such as geographic remoteness, that is designed to predict economic status [15]. Several studies have suggested that the test misses many of those in poverty, however, partly because it focuses too much on emulating income measures of deprivation [16].

The paper aims to derive an index of urban poverty that reflects both chronic and acute vulnerability from available data. It is structured as follows. The next section describes the data used and methods adopted in the analysis of poverty including a participatory measure of socio-economic status based on a variety of qualitative data collection methods that we use as a way of synthesising our understanding of poverty. We then present results focusing first on existing measures followed by findings from the participatory qualitative assessment and a new indicator based on the participatory assessment that captures the acute and chronic nature of poverty. The final section discusses the implications these methods have for future assessment of vulnerability used to determine access to various services in urban settings.

## Materials and methods

We utilise data collected as part of the Surveys for Urban Equity (SUE) project that focuses on household health survey methods in urban areas of rapidly urbanising countries of Asia: Nepal, Bangladesh and Vietnam. This study specifically uses the data set from the household survey in Nepal which was designed to be representative of the Kathmandu Valley [17]. A total of 1,180 households which include 4,483 individuals from 60 randomly selected primary sampling units (clusters) were surveyed between November 2017 and January 2018. Members of the household were asked to identify the person most knowledgeable about the household including incomes and spending decisions. This person was then interviewed to obtain information on household structure and characteristics, migration, social capital, income and consumption expenditure and also had specific individual modules on mental health (depression) and injuries.

Using data on households and some individual questions from the SUE survey, we construct a series of indices that attempt to assess levels of vulnerability at the household level. In total, seven different measures are calculated (Table 1). These measures are of four types. Firstly, those that focus on immediately available resources that can be used to construct monetary poverty lines: income per capita [1] and consumption per capita [2]. Second, the asset index [3], pioneered in the analysis of DHS [11], focuses on physical wealth or capital. The third group combines both physical and human capital into a multidimensional measure of wealth status, which include here the Progress out of Poverty Index (PPI) [4] [18], the Deprivation index [5] [3] and the UN Habitat slum index [6] [19]. Finally, self-poor [7] is based on a survey question asking the head of households how they judge their own living standards relative to others in the community.

**Table 1:** Measures of vulnerability computed from household data.

	Measure	Type	Description
1	Income per capita	Current resources	Income received by any member of the household from any source including: daily labouring, monthly salary, rent, investments, loan interest, self-employment (next of expenses), agriculture, retirement and other state benefits and asset sales. Income is annualised for each source, aggregated to the household level and calculated by household member (per capita).
2	Consumption per capita		Total spending by household on: items purchased frequently over the last 30 days including food and non food items such as utilities, rent, education and health care; and items purchased infrequently over the last 12 months purchased including furniture, electronic goods and transportation. Values are imputed for crops and other items produced by the household or gifted to the household. Spending is annualised and calculated per household member. Food spending is adjusted for different purchasing power across clusters. A volume weighted index was derived based on the price of the top 10 food items available the closest market/food store in each cluster.
3	Asset index	Physical capital	Index constructed from a principal component analysis (first component) of household assets covering availability of water and sanitation, type of cooking fuel, size of housing and construction materials, ownership important consumer durables (e.g. computer, refrigerator, mobile phone), ownership and livestock and land. All variables used to construct the asset index in the 2016 Nepal Demographic and Health Survey are included.
4	Progress Out of Poverty (PPI)	Physical and human capital	The progress out of poverty indicator is a country-specific index developed by progressoutofpoverty.org and based on multivariate analysis of the determinants of household income and consumption. In the Nepal version there are ten indicators: 1) number of household members; 2) job worked by head of household or spouse; 3) bedrooms available; 4) construction of walls and 5) roof of dwelling; 6) availability of a kitchen; 7) type of stove used; 8) type of toilet; 9) number of telephones in household; and 10) whether land owned for agriculture. Points are assigned to each question depending on response with a maximum score (no poverty) of 100.
5	Deprivation		The deprivation index based on Bag and Seth 2017 examines household access to a range of functions. The index used in the paper has 11 items. Our survey allows us to calculate nine of these: access to improved water source, sanitation, structure of house, level of over-crowding, respiratory health risk from cooking stoves, access to saving instruments, asset ownership, access to phones and education attainment.
6	UN Habitat slum		UN Habitat index of whether a household is classified as an informal (slum) settlement. It includes: housing wall construction, household overcrowding (number of people sharing each room), availability and cost of water and availability and type of toilet.
7	Self Poor	Self-defined	Based on whether a household (household head) self-defines as much or slightly poorer compared to other households in the community.

We compare the level of agreement across the indices. For selected indices, the characteristics of those falling into the lowest quintile (”poor”) are compared with those in other quintiles (“non-poor”), and these differences in characteristics are compared across indices to see how they agree or vary depending on the correlation between the indices. Consumption is chosen as an indicator of immediate ability to obtain necessities (acute or immediate poverty) given that it is generally easier to assess than income which is the other main method of understanding current resources. The deprivation index used as representative of a class of multi-dimensional-asset indicators indicative of chronic, capital-stock based poverty. The deprivation index is chosen because it incorporates most of the dimensions included in the other capital focused indices. Sub-dividing the sample by migration status enables a comparison between the types of poverty represented in each group given that initial analysis suggested that migration status substantially affected the nature of wealth and poverty. All analyses were performed in Stata v15 and applied sampling probability weights and adjusted for clustering.

The second section of the study used participatory methods within urban neighbourhoods to generate qualitative information on differences in poverty. The advantage of this process is that local intelligence on what leads to and constitutes poverty can be used to assess poverty [20]; in this case the specific context of a poor area with informal settlements. Rather than asking which households are poor in an area, a process that can lead to capture by vested interests and does not lead to elicit a tool that is transferable across communities [21] we seek information on the characteristics of households considered poor and non-poor. This information can then be used to prioritise across the huge range of possible variables in the construction of the index.

Two areas in Kathmandu were selected due to their varying characteristics; one was an informal settlement and the other a more mixed neighbourhood with pockets of poverty [17]. Within the two areas a series of participatory methods including social mapping, transect walk, photovoice and wealth ranking were used with community members, purposively sampled to create a maximum variation sample by age, sex, wealth and caste (S1 Annex). In each site community leaders were asked to identify participants across the age, sex, wealth and caste spectrum in their area. Our researchers then approached these individuals and explained an information sheet about the study verbally and left a Nepalese copy for each participant to decide whether to consent to participate. Those consenting then met the research team at the allotted time to participate in the first method, social mapping. Data collection was led by two experienced Nepalese qualitative researcher SM and SK. Training on the participatory methods was developed by HE who has extensive experience of participatory methods and qualitative methods. A further three data collectors were involved and they all received training on the methods from SM and SK. Training included using the methods with a group of volunteer community members, who provided feedback and advice on how they could be improved. Following use of the methods in one community, the team reflected on the process and made slight variations in subsequent data collection, such as including involving different community members in the different methods to gain a greater variety of insights. During data collection, researchers kept reflective logs on the process and observations of the interactions between community members. This enabled careful facilitation to ensure that quieter participants were encouraged to contribute during the methods. This and the use of community leaders to help with recruitment of participants of differing ethnicity, gender, age, occupation, caste helped to reduce any bias in the data collected.

During the social mapping, participants mapped their neighbourhoods by hand sketching roads, dwellings and any landmarks on paper. During the transect walk, small groups of participants (7 in site one and 4 in site 2) agreed a route within the area represented in the social map, that would show different levels of poverty in their neighbourhood. Whilst drawing the maps and taking the transect walk, the researchers facilitated discussion about the poverty and vulnerability of different households. For photovoice data collection, participants took photographs (with consent) of the dwellings and compounds, including their own, that they felt best displayed the varying levels of poverty and vulnerability of households in their neighbourhood. Finally, in the wealth ranking exercise participants were asked to assign different households to one of three categories: very poor, not too poor and better off/more resilient. Throughout, the researchers probed to explore why participants placed certain households in each category. All discussions held during the social mapping, transect walk, and wealth ranking exercises were recorded and transcribed into English. The photographs, along with the transcripts, were analysed using a framework approach [22, 23], with the data managed in NVivo 11. The analysis followed five main steps including i) familiarisation with the transcripts; ii) identifying an initial framework based on the poor, not-so poor and better off categories identified by the communities; iii) codes were then developed from the data showing the different factors communities identified as influencing poverty/wealth; iii) the coded text was then transferred into charts under each of the categories; iv) the final step involved mapping the linkages and contrasts between the factors and particularly identifying any intersectional gender differences in perceptions on poverty and wealth. These steps were undertaken initially by Nepali research SM. HE and ANP blind-coded three transcripts and compared coding with SM. This process has been recommended as a way to reduce bias within the analysis process [24]. This led to the development of the coding framework. The mapping stage (v) was conducted during a team workshop where all researchers involved were able to draw out deeper insights into the factors influencing vulnerability to urban poverty. These detailed findings will be presented in a future paper.

The qualitative and quantitative study proposals, instruments, information sheets and consent procedure were approved by the ethics boards of the University of Leeds, School of Medicine (SoMREC Ref: MREC16-137) and Nepal Research Council (NHRC) Ref no. 191/2017). Verbal consent was obtained from participants by reading out the information sheet to potential participants and if consent was indicated the interviewer then ticked a box on the questionnaire to indicate implied consent. During piloting we found that gaining written or thumb-print consent (as originally planned) was problematic with potential participants explaining that while they were willing to do the questionnaire interview, they were nervous to sign any documents. This was a particular concern in low-income and informal settlements, so without this change to the ethics procedure we were concerned that we might have issues with recruitment bias. An amendment to our ethics procedure was approved by the University of Leeds. Only participants 18 and over were interviewed for both studies.

The third section of the study makes use of the qualitative findings to derive an index that incorporates both indicators of longer-term, chronic poverty and immediate measures of resource accessibility associated with the vicissitudes of urban living. The indicators derived from the qualitative data are matched with similar variables from the quantitative analysis to derive a composite measure that combines both productive and consumption assets with measures of current resource availability. Subsequently, we investigate whether a smaller subset of variables might be used to identify households falling into poverty without collecting full information on consumption, asset and other household vulnerabilities. A subset of variables were used in this process which were reduced through stepwise logit regression. The overall sensitivity (proportion of the poor identified as poor) and specificity (proportion of those that are identified as poor that are actually in poverty) of the resulting indicator is computed. Receiver Operator Characteristic (ROC) curves are plotted which calculate sensitivity and specificity of the measure at different poverty thresholds. All data are analysed using Stata (15 including the downloadable ado post-estimation procedures lroc and roccomp to calculate the ROC curves.

## Results

**Fig 1:**
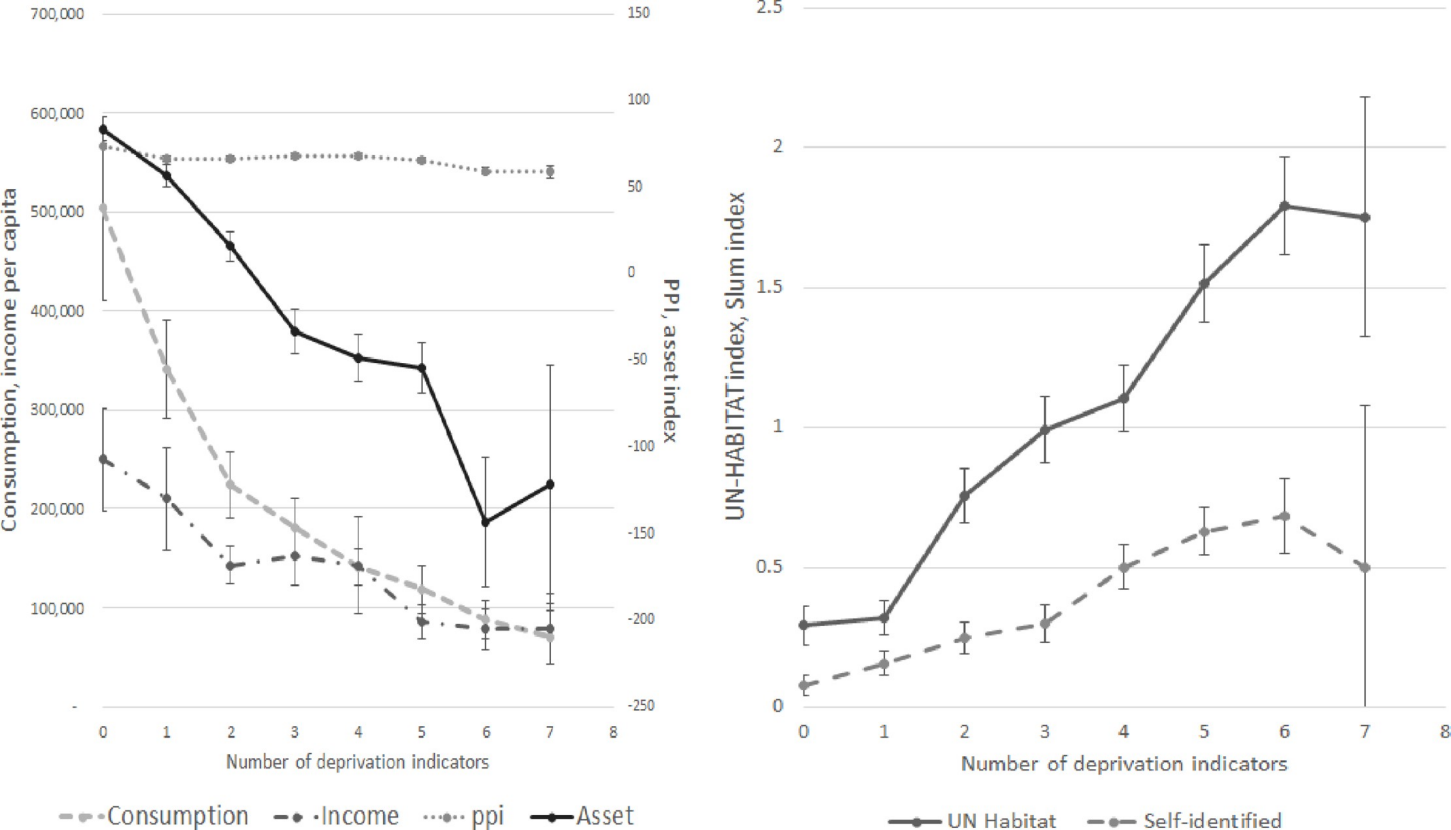
a & b Relationship of deprivation Index with other vulnerability indices.

Most of the indices show similar changes across the population. Taking deprivation [5], as a reference index, which ranges in value from 0 (low deprivation) to 7 (high deprivation), most of the indicators that are designed to increase with socio-economic status—consumption, income and assets–fall as the number of deprivation indicators recorded by households increase (Fig 1A). The exception is the PPI index which is more or less unchanged across a number of groups; the change in the index between most and least deprived is only 21% compared to a variation of 80% or more for other indicators. Income and assets, although declining overall, shows some fluctuation in the mid-range. This could be due to the problems with measuring income but may also reflect the differences in chronic and acute manifestations of poverty. The three indicators that are positively related to deprivation mostly increase across the deprivation score although self-identified poverty flattens off (Fig 1B).

**Fig 2:**
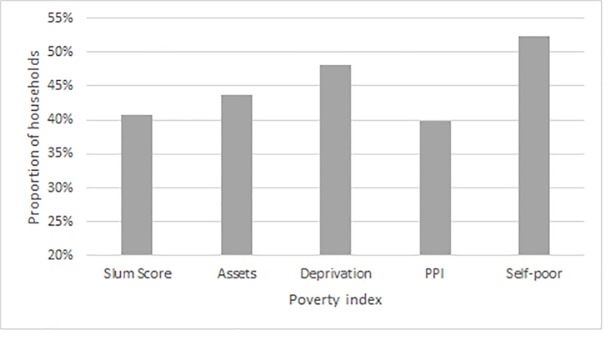
Proportion of the consumption poor identified using each poverty index.

Although there is general agreement between indices, the asset and capital based measures only partly identify households that fall below an absolute monetary measure of poverty. Around 17% of households were defined as absolutely poor based on current resources assessed by consumption spending. The asset, capital and self-defined measures of poverty identify between 40% and 60% of those households falling below a monetary poverty line (Fig 2). These measures prioritise different aspects of poverty, particularly associated with various assets which will be important determining longer term economic status. While the asset and multidimensional indices capture medium to longer term aspects of resources and capabilities that suggest chronic poverty, they are less able to identify immediate cash constraints that are important particularly in urban contexts where alternative non-cash resources from, for example, subsistence farming are not available. Merging both multidimensional asset and consumption indices could help to capture both chronic and short-term, acute aspects of poverty into a single assessment.

The differences between the poverty measures can be further understood by looking at the characteristics of those defined as poor or non-poor using the deprivation and consumption measures. Comparing the consumption (“acute poverty”) and deprivation (“chronic poverty”) index four groups are defined as households that are: 1) non-poor under both measures; 2) poor using deprivation, non-poor using consumption; 3) consumption poor, deprivation non-poor; and 4) poor under both measures. Groups 1 and 4 might be described as unambiguously non-poor or poor and more than 80% of households in our sample fall into one of these groups (71% non-poor, 9% poor).

A number of characteristics distinguish groups 1 (non-poor) and 4 (poor). The non-poor are much more likely to have a room to rent out, have completed secondary and primary education and own farming land (Table 2). The poor (group 4) are more likely to have been evicted and spend a much larger proportion of their financial resources on rice, the main primary food commodity (8% versus 3%).

**Table 2:** Characteristics of households in the bottom quintile (“poor”) versus other quintiles (“non-poor”) of the consumption and deprivation indices (means and 95% confidence intervals).

	1. Non poor	2. Dep poor, cons non poor	3. Cons poor, dep non-poor	4. Poor
% of consumption on rice	3.2%	6.0%	6.8%	7.6%
	(2.96% , 3.36%)	(5.29% , 6.81%)	(5.77% , 7.84%)	(6.59% , 8.67%)
% consumption on 12 food items	16.1%	26.7%	32.9%	83.3%
	(15.21% , 16.98%)	(23.68% , 29.80%)	(24.59% , 41.19%)	(35.97% , 130.62%)
% of hh dependents	20.0%	26.5%	24.5%	29.1%
	(18.45% , 21.50%)	(21.37% , 31.54%)	(20.18% , 28.72%)	(24.53% , 33.65%)
Female household head	24.3%	32.6%	33.6%	28.9%
	(21.39% , 27.26%)	(22.59% , 42.58%)	(25.02% , 42.19%)	(20.41% , 37.48%)
Whether migrated	66.8%	89.9%	66.4%	80.7%
	(63.56% , 70.01%)	(83.46% , 96.32%)	(57.81% , 74.98%)	(73.28% , 88.13%)
Migrated out of economic necessity	31.1%	67.4%	46.7%	62.3%
	(27.94% , 34.28%)	(57.42% , 77.41%)	(37.65% , 55.79%)	(53.16% , 71.40%)
Migrated within last 5 years out of economic necessity	6.3%	16.9%	6.6%	22.8%
	(4.65% , 7.98%)	(8.87% , 24.83%)	(2.06% , 11.06%)	(14.91% , 30.70%)
Whether been evicted	0.9%	4.5%	0.8%	6.1%
	(0.28% , 1.59%)	(0.08% , 8.91%)	(-0.82% , 2.46%)	(1.62% , 10.66%)
Whether own house	45.7%	2.2%	34.4%	8.8%
	(42.32% , 49.14%)	(-0.91% , 5.41%)	(25.79% , 43.06%)	(3.45% , 14.09%)
Own agricultural land	61.3%	74.2%	63.1%	69.3%
	(57.95% , 64.62%)	(64.82% , 83.49%)	(54.34% , 71.89%)	(60.62% , 77.98%)
HH completed primary education	82.0%	48.3%	61.5%	45.6%
	(79.36% , 84.62%)	(37.66% , 58.97%)	(52.63% , 70.32%)	(36.24% , 54.98%)
HH com[leted secondary education	40.5%	21.3%	29.5%	19.3%
	(37.11% , 43.83%)	(12.61% , 30.08%)	(21.22% , 37.80%)	(11.87% , 26.72%)
Self defined as poor	20.6%	56.2%	43.4%	64.0%
	(17.82% , 23.35%)	(45.60% , 66.76%)	(34.43% , 52.46%)	(55.01% , 73.06%)
Has room to rent	29.4%	0.0%	9.0%	1.8%
	(26.24% , 32.47%)	(0.00% , 0.00%)	(3.81% , 14.22%)	(-0.72% , 4.22%)
Households receiving renittances	14.7%	11.2%	6.6%	7.0%
	(12.31% , 17.16%)	(4.50% , 17.97%)	(2.06% , 11.06%)	(2.21% , 11.82%)
Unemployed head of household	10.5%	1.1%	2.5%	6.1%
	(8.43% , 12.63%)	(-1.12% , 3.37%)	(-0.36% , 5.27%)	(1.62% , 10.66%)
Percentage of houshold members unemployed	26.5%	10.1%	29.5%	18.4%
	(22.76% , 30.34%)	(2.25% , 17.97%)	(16.40% , 42.61%)	(10.71% , 26.13%)
Income per capita	205,819	124,867	86,109	61,455
	(18278813.04% ,	(10036698.74% ,	(6939361.67% ,	(4855531.71% ,
Average age of household members	32.04	24.27	29.92	26.78
	(3124.75% ,	(2229.34% ,	(2800.66% ,	(2441.88% ,
Household size	3.60	3.30	4.32	3.55
	(347.78% , 373.16%)	(302.22% , 358.46%)	(394.70% , 469.23%)	(327.28% , 383.25%)
Number employed	1.22	1.20	1.42	1.05
	(114.89% , 128.38%)	(96.70% , 143.75%)	(124.56% , 159.04%)	(86.31% , 124.22%)
Social capital [1]	2.18	1.82	2.17	1.81
	(211.21% , 225.04%)	(161.42% , 202.63%)	(199.08% , 235.35%)	(160.94% , 200.46%)
Sample size	855	89	122	114
% of total sample	72%	7.5%	10.3%	10%

1. The social capital score is a simply summation of affirmative (= 1) responses to the questions whether households trust community members, don’t have to be alert to other taking advantage, agree that community members are willing to help out if needed, trust community members in matters of lending and borrowing money. HH = household.

Households that are classified as consumption poor but not deprived (group 3) spend a similar proportion of their income on rice as the poor, are unlikely to receive remittances or have a room to rent. They are however more likely than the poor to have received primary education and own a house. It is also notable that more than 90% of households in group 3 are both absolutely as well as relatively poor, spending less than $1.9 (NR 216) per capita, per-day national poverty line [25]. This group, therefore, appears to be historically non-deprived but overwhelmingly acutely poor and unlikely to be able to afford essential items possibly as a result of recent adverse circumstance.

Households classified as deprived but not consumption poor (group 2) spend less than the consumption poor on basic foods such as rice. They are less likely to be educated to a primary or secondary level or own a house, have lower levels of social capital and none of the households in this group have a room to rent. The majority of this group are in the Kathmandu Metropolitan Area (KMA): while 4% [26] of households outside KMA are in group, this rises to 8% (n = 46) for those inside KMA (Table 3). While they may not be immediately poor, the lack of assets and access to other variables suggests that they are vulnerable to economic shocks.

**Table 3:** Migration status of households by vulnerability category (number of observations and 95% confidence intervals in brackets).

	By migration status				By residence	
	Non-migrant	Migration for other reasons	Forced to migrate for economic reasons		Kathmandu	Other
1. Non-poor	79.4% (n = 459)	79.7% (n = 245)	54.4% (n = 160)		67.9% (n = 392)	77.0% (n = 464)
	(68.6% , 87.2%)	(68.8% , 87.5%)	(43.6% , 64.8%)	,	(56.8% , 77.3%)	(69.5% , 83.1%)
2. Deprivation poor, consumption non poor	1.8% (n = 10)	4.6% (n = 14)	12.3% (n = 36)		8.0% (n = 46)	4.0% (n = 24)
	(0.8% , 3.8%)	(2.5% , 8.6%)	(8.3% , 18.0%)	,	(5.3% , 11.8%)	(2.2% , 7.4%)
3. Consumption poor, deprivation non-poor	11.8% (n = 68)	8.8% (n = 27)	13.6% (n = 40)		12.2% (n = 71)	9.1% (n = 55)
	(6.4% , 20.9%)	(4.4% , 17.0%)	(9.6% , 19.0%)		(7.8% , 18.7%)	(5.2% , 15.4%)
4. Poor (consumption & deprivation)	7.0% (n = 40)	6.8% (n = 21)	19.7% (n = 58)		11.9% (n = 69)	9.8% (n = 59)
	(4.0% , 11.9%)	(3.9% , 11.7%)	(11.9% , 30.6%)		(6.9% , 19.8%)	(6.1% , 15.4%)
Number of households	578	308	294		578	602

Households that have migrated to Kathmandu for reasons other than economic necessity–including education, to join other family member or for better housing—are no more likely to be poor than those that have lived in the city for generations (Table 3). This can be contrasted with those moving out of economic necessity; these households are more than twice as likely as non-migrants to be consumption poor and deprived (19.7% compared to 7%) and more likely to be defined as deprived but not consumption poor (12.3% compared to 1.8%).

The data suggests differences in the socio-economic profile of migrants and non-migrant households (Table 4). Where available, information for all urban residents from the Nepal 2016 DHS is provided for comparison. Migrants, both economic and other, are less likely to own assets such as a fridge, TV or motorbike, than non-migrants reflecting their less settled status and acquisition of wealth. Migrants are more likely to have access to farmland although not to farm animals. Non-migrants are, however, much more likely to own valuable land in the Kathmandu valley. Migrants and non-migrants have similar access to an improved water source but non-migrants are more likely to rely on bottled rather than improved water sources. Migrants are also more likely to have a piped toilet. Both these findings may reflect the greater likelihood that migrant families live in communities with more recently developed amenities. It should also be noted, however, that bottle water is often used as a cleaner alternative to piped water and so use remains a measure of wealth.

**Table 4:** Wealth characteristics of migrant and settled population.

	DHS 2016	SUE Survey 2018								
	Urban	All	95% CI	Non- migrant	95% CI	Migration for other reasons	95% CI	Forced to migrate for economic reasons	95% CI	Diff. (non & econ migrants)
***Household assets***										
Radio	28%	18%	[15.9%, 20.2%]	25%	[20.8%, 29.8%]	19%	[14.9%, 22.9%]	12%	[8.7%, 14.6%]	14%
Refrigerator	22%	49%	[46.0%, 51.8%]	72%	[67.2%, 76.6%]	44%	[39.0%, 49.1%]	35%	[30.4%, 39.2%]	37%
Fans	54%	33%	[30.6%, 36.0%]	45%	[40.0%, 50.4%]	29%	[24.6%, 33.8%]	27%	[23.2%, 31.4%]	18%
TV	62%	79%	[76.6%, 81.2%]	95%	[92.7%, 97.2%]	65%	[60.6%, 70.3%]	77%	[73.5%, 81.2%]	18%
Telephone—mobile	94%	98%	[96.7%, 98.4%]	99%	[97.8%, 100.0%]	98%	[96.0%, 99.1%]	96%	[94.8%, 98.2%]	2%
Computer/printer	18%	39%	[36.5%, 42.0%]	49%	[44.0%, 54.4%]	48%	[42.5%, 52.7%]	25%	[20.7%, 28.6%]	24%
***Transport***										
Bicycle	39%	12%	[9.8%, 13.4%]	14%	[10.7%, 18.0%]	7%	[4.6%, 10.0%]	13%	[9.9%, 16.1%]	1%
Motorcycle	23%	40%	[37.6%, 43.2%]	57%	[52.2%, 62.4%]	36%	[31.6%, 41.4%]	30%	[26.2%, 34.6%]	27%
Car	4.3%	7%	[5.3%, 8.2%]	10%	[6.5%, 12.6%]	7%	[4.6%, 10.0%]	4%	[2.3%, 6.0%]	5%
***Household characteristics***										
Use bottle water	4.4%	53%	[50.4%, 56.1%]	38%	[32.6%, 42.7%]	61%	[55.8%, 65.8%]	59%	[54.7%, 63.8%]	-22%
Improved water source	93%	96%	[94.6%, 96.9%]	97%	[95.8%, 99.1%]	98%	[97.1%, 99.7%]	92%	[89.8%, 94.7%]	5%
Piped toilet	7%	73%	[70.1%, 75.2%]	61%	[56.2%, 66.3%]	79%	[75.0%, 83.3%]	76%	[72.3%, 80.1%]	-15%
Improved toilet	61%	97%	[95.8%, 97.8%]	99%	[97.8%, 100.0%]	96%	[94.6%, 98.4%]	95%	[93.4%, 97.3%]	4%
LPG for cooking	46%	97%	[95.7%, 97.7%]	96%	[93.4%, 97.7%]	99%	[98.3%, 100.1%]	96%	[93.7%, 97.5%]	0%
Clean fuel for coooking	70%	97%	[96.0%, 97.9%]	96%	[94.0%, 98.1%]	99%	[98.7%, 100.2%]	96%	[93.7%, 97.5%]	0%
Cooking in house	70%	97%	[96.5%, 98.3%]	98%	[96.6%, 99.5%]	99%	[98.3%, 100.1%]	95%	[93.4%, 97.3%]	3%
Has electricity	94%	99%	[98.4%, 99.6%]	100%	[99.2%, 100.3%]	100%	[99.2%, 100.3%]	98%	[96.4%, 99.1%]	2%
Own farmland		63%	[63.2%, 63.2%]	40%	[34.5%, 44.7%]	73%	[68.7%, 77.8%]	74%	[69.5%, 77.6%]	-34%
Own land in Kathmandu valley		11%	[9.3%, 12.9%]	28%	[22.9%, 32.2%]	5%	[3.1%, 7.7%]	3%	[1.3%, 4.4%]	25%
Own farm animals	58%	6%	[4.7%, 7.5%]	14%	[10.2%, 17.3%]	2%	[0.7%, 3.6%]	3%	[1.7%, 4.9%]	10%
Room to rent		22%	[20.0%, 24.8%]	40%	[34.8%, 45.0%]	21%	[16.4%, 24.7%]	10%	[7.4%, 12.9%]	30%
Household size	4.39	3.65	[3.7 , 3.7]	4.14	[3.9 , 4.3]	3.25	[3.1 , 3.4]	359%	[3.4 , 3.7]	55%

Analysis of a range of poverty measures suggests they produce overlapping but different assessments of poverty. In particular, multi-dimensional asset measures that are generally favoured as measures of long term, chronic poverty fail to identify a significant minority of households that lack immediate resources for basic needs. This is particularly important in urban areas since opportunities to supplement basic food needs are lacking. Across the sample, the value of own production, both food and non-food, constituted less than 0.96% of total consumption rising to 2.8% for the poorest 20% of households. This contrasts with national figures based on the Nepal Living Standards Survey 2011 of 3.6% for households in rural areas and 5.7% for the poorest quintile [26]. This suggests that assessment of socio-economic status needs to include both measures of physical and human capital and immediate access to resources. These issues are explored through the qualitative analysis.

The analysis of the participatory qualitative data identified eleven domains over which individuals distinguish between poor, medium and the better-off: house structure and environment; household assets; occupation; income related; business; education; land holdings and ownership, healthcare; basic needs, means of transportation and social capital (Table 5).

**Table 5:** Main features of poor, medium and better-off households from qualitative data and proxy quantitative indicators from household survey.

	*Main differences between groups*		
	Rich	Poor	Variables chosen
1. Housing	House related: Type of house construction, size & internal decoration	Temporary construction, small dwelling	Produc tive asset: 1. House construction materials; 2. number of rooms in the house; 3. spending on rent
2. Assets	Ownership of relatively new household assets (e.g. fridge, TV, washing machine)	Few new assets	Consumption asset: 4. Fridge; 5. TV; 5. no spending on durables in last 12 months
3. Occupation	Regular, salaried skilled job	Irregular, unskilled or no employment	Current resources: 6. Head of Household is unskilled labourer; 7. seasonal labourer; 8. ratio non-earners: earners (>2)
4. Income	Substantive, multiple incomes, few dependents	Few earners, large number of dependents	Current resources: 9. consumption per capita ; 10. No one working in the household
5. Business	Well established business	No business or informal business	Produc tive asset: 11. Ownership of a business
6. Education	Most household members well educations	Few or no one with schooling	Produc tive asset: 12. No one with schooling in household
7. Land	Ownership of land	No land owned	Produc tive asset: 13. Do not own high value land in Kathmandu valley
8. Basic	Good food, water	Unable to meet	Current resources: 14. Do not use
needs	and clothing	basic food needs	water from jar/direct supply of water
	available	and lacks access	to home;
		to potable water	15. % spending on rice
9. Health care	Able to afford treatment when required	Finds it difficult to pay for medical care	Current resources: 16. Catastrophic health spending—defined as a household spending more than 10% of consumption on health care
10. Transport	Own motor transport	No transport, must walk or take the bus	Consumption/produc tive asset: 17. Do not own motor transport (car/motorcycle)
11. Social capital	Close involvement/links to support household	Few/no available family or community links to offer support to household	Produc tive asset: 18. Low social capital defined as households that are not members of community groups or lack trust in community members.

A more detailed table can be found in S1 Annex

The participatory work identified a range of factors that distinguish well-off from medium and poor households. This includes variables that are descriptors of longer term or chronic poverty: both i) productive assets (domains 1, 5, 6, 7, 12, 13, 18) that help enhance livelihoods such as education and ownership of land [27] and ii) consumption assets [2, 11, 17] that largely suggest that a household has earned or inherited wealth in the past. It also includes iii) indicators of current resources and ability to meet basic needs (domains 3, 4, 8, 9, 10, 14, 15 & 16) such as consumption spending, present occupation and use of potable water sources for drinking. These provide an indication of shorter-run movements in and out of poverty resulting from sickness, population movement or change in employment [28].

For each domain described by participants, a quantitative indicator was identified in the household survey dataset. Using the quantitative variables identified in the participatory assessment on current resources and physical and human capital enables the construction of a combined, context specific Urban Poverty Index (UPI) that incorporates both chronic and acute aspects of household socio-economic status. In a few cases a direct proxy for the indicator was not available and an indirect proxy was used. This includes: lack of spending on assets in the last 12 months as a proxy for named assets that are considered old; labourer/unskilled job status as a proxy for a cluster of occupations associated with poverty and high rice consumption as a % of total spending as a proxy for a lack of ability to properly feed children in the family. Households are categorised by analysing access to assets and employment states and then consumption data as follows:

$$UPI\;(1/0)=if\;R_{20}\;\left\{\frac{1}{n}\sum_{i}^{n}\Phi_{i}\right\}\;or\;(cons_{pc}<\pi )$$

where Φ_*i*_ are a series of *n* binary variables that describe whether the household has access to physical and human capital (Table 5) , R_20_ represents the bottom quintile of this capital distribution and *cons_pc_* is per capita annual household consumption adjusted for geographic differences in prices of essential goods and *π* is the (annual) poverty line. A household is defined as “poor” if they either fall into the bottom quintile for access to assets and employment or have consumption below the national poverty line. The Government of Nepal uses the international poverty line of $1.9 converted into Nepali Rupees, which for the purposes of this analysis is taken to be Rs 212 per day [29]. The resulting index identifies 31% of households in the sample areas as poor (Table 6). This is close to national level figures on poverty such as those of the recent Multi-dimensional Poverty Index that found that ‘28.6% of Nepal’s population is multi-dimensionally poor’ [25]. Assets, consumption and income per capita are clearly different across the two groups. Just over 61% of the poor group are identified as in the lowest consumption quintile and 41% as in the lowest quintile using the deprivation index compared with 2.4% and 7.1% respective in the non-poor group. This suggests that the index is generally successful in mapping to both capita/chronic and consumption/acute measures of poverty.

**Table 6:** Characteristics of sample using Urban Poverty Index.

	Sample size	Consumption per capita	Income per capita	Asset index	Primary education	Consumption Q1 (%)	Deprivation Q1 (%)
Non-poor	813	314,474	188,801	0.31	79.6%	2.4%	7.1%
%/CI	69%	(287,248 , 341,699)	(168,123 , 209,479)	(0.26 , 0.36)	(77% , 82%)	(1.4% , 3.5%)	(5.4% , 8.9%)
Poor	367	108,601	107,949	- 0.69	61.0%	61.5%	41.0%
%/CI	31%	(93,038 , 124,163)	(86,802 , 129,096)	(-0.81 , -0.56	(56% , 66%)	(56.4% , 66.6%)	(35.9% , 46.2%)
Total	1,180	250,444	163,655	0.00	74%	20%	17%
%/CI	100%	(230,326 , 270,561)	(147,823 , 179,486)	(-0.06 , 0.06)	(71% , 76%)	(17.7% , 22.3%)	(15.0% , 19.4%)

A problem with the UPI is that it requires an assessment of consumption levels. The final part of the analysis seeks to assess whether the index can be simplified without substantial loss of precision using variables that are more easily measured. We used stepwise, logistic regression to reduce the number of variables used to categorise households into poverty groups. Two models are estimated. The first model includes simple measures of proportionate spending on rice and rent which together explain more than 75% of the variance in consumption spending across the sample together with physical and human capital variables. Given the association between patterns of poverty and migration we also include economic migrant status in the model. The second model leaves out the consumption variables and substitutes these for questions on employment status, household members and physical capital.

The model with rice and rental spending lead to a prediction model with 10 coefficients significant at least at the 1% level (Table 7). The sensitivity and specificity of the model varies according to the prediction threshold to define a household as poor; a test sensitivity of 81% implies here specificity of 80% (where sensitivity is defined as one minus the probability of a false negative; specificity as one minus the probability of a false positive. With any diagnostic test, there is generally a trade-off between these two measures). The area under the ROC curve (AUC), which is a bounded index ranging from 0 to 1, where 0.5 means the test is no better than chance and 1 means perfect identification, is 0.89. Model two excludes the less easy to measure variables: proportion spending on rice and rental spending. The step-wise procedure adds roofing material of dwelling, migration out of economic necessity, no one educated beyond primary level and ownership of a fridge. The AUC falls very slightly to 0.87. The results suggest that a narrower range of variables can be used to identify the urban poor but with some modest loss in precision.

**Table 7:** Predictors of poverty status [1].

	All Characteristics			Without consumption predictors		
Variable	Coef	SE		Coef	SE	
How many people are in your household?	0.2012	0.0591	***	0.1498	0.0558	***
Roof of dwelling made from straw, mud or wood planks OR walls made of bamboo/leaves or mud-bricks or stones.	-	-	***	0.8288	0.3908	**
More than 3 people sleeping in each room.	0.6306	0.1934	***	0.8012	0.1894	***
Household does not have a television.	1.0223	0.2111	***	1.0610	0.2138	***
Have you migrated to this area for reasons for economic necessity?	-	-	***	0.2935	0.1764	*
HH does not own a business	2.1034	0.3739	***	2.2421	0.3771	***
No one educated beyond primary school in household	-	-	***	2.6247	1.1756	**
No access to (potable) jar water.	1.4564	0.1949	***	1.3517	0.1889	***
No access to own motorised transport (motorbicycle or car)	1.6219	0.2232	***	1.5657	0.2208	***
No one working in the household	0.5125	0.2116	**	0.6085	0.2098	***
Did the HoH complete primary education?	-	-	***	- 0.3394	0.1920	*
Did the HoH complete secondary education?	- 0.4763	0.1821	***	-	-	***
How much does your household spend on rent per month?	- 0.0001	0.0000	***	-	-	***
Does not own a fridge	-	-	***	0.3889	0.2218	*
What proportion of your consumption is spent on rice?	9.1861	2.2343	***	-	-	***
Do you have a room available to rent to others?	-	-	***	- 0.6133	0.2917	**
Do you consider your household poor compared to your neighbours?	-	-	***	0.4115	0.1802	**
Constant	- 5.1516	0.5473	***	- 5.7577	0.5768	***
n	1,180			1,180		
pseudo R2	0.39			0.35		
AUC	0.89	0.01		0.87	0.01	

significant at the *** 1% level, ** 5% level, * 10% level.

1. Based on a stepwise regression. Full list of variables included in the step-wise regression: gender of head of household (HoH), whether evicted, migration for economic reasons, migration in last 6 months, not owning land in Kathmandu valley, little/no education of HoH, HoH completed primary/secondary/higher education, self-defined as poor, whether has room to rent, receipt of remittances, HoH unemployed, rice/rent as % of consumption, household size, age of HoH, owning a business, access to jar water, not owning motorised transport, roof/wall materials of dwelling, number of members sleeping per room, seasonal working, ownership of assets (TV, fridge)

## Discussion

Drawing on a large quantitative dataset and a participatory community study we have generated an urban poverty index that captures both aspects of socioeconomic status based on information captured by a large survey of households. This index is then simplified by identifying a smaller group of easily measured variables. This simplification leads to some loss of specificity but has the advantage that far less information is required on household status particularly on consumption spending.

Our assessment of poverty is underpinned by a qualitative participatory analysis of the factors that identify households as poor and non-poor that is then linked to measurable quantitative variables in a single index. Combining these approaches helps to ensure that the index is driven by local experience of poverty that can assessed in a quantifiable way. Most indicators of poverty either focus on immediate money resources (consumption, income) or physical and human capital stocks. They are often viewed in an interchangeable way with one as a proxy for the other. Even multi-dimensional poverty indicators used internationally including in Nepal tend to focus on assets and human capital [3, 25]. Our participatory analysis suggests that the measurement of urban poverty needs to take account of physical and human capital (chronic poverty) and also short-term, cash or immediate resources (acute poverty) that better describe acute poverty in order to understand urban economic vulnerability. While there are some households that are assessed as poor using both measures, a substantial minority of the population (19% in this sample) are ambiguously classified. Around 45% of these are above the poverty line but are likely to remain economically vulnerable because they lack assets. They are also more likely to have recently migrated and so are less likely to have strong social networks to support them through economic shocks.

Consumption and income-based indicators of poverty certainly have limitations in their ability to properly describe economic opportunities and other aspects of well-being. Yet leaving out consumption indicators runs the risk of failing to properly target groups that lack disposable resources to purchase everyday basic needs. Cash resources are perhaps more important in urban areas resulting in the need for an index that incorporates current resources into the overall assessment A significant minority of households (11%, Table 2) are defined as non-poor when consumption is excluded despite falling below the absolute international poverty definition of around $2 per capita/day.

Developing a fast but reliable method of assessing urban poverty status is important for a whole range of targeted programmes but is not unproblematic. Choosing a shortlist of easily measurable items have been shown, for example in the case of the PPI measure, to accurately predict poverty in the short term but less accurate longer term as the items chosen become weaker tracers of wealth [18]. Easily measurable items also tend to assess indicators that are more indicative of longer term wealth and may not pick up poverty that is related to shorter-term monetary factors that also push households into poverty. Our analysis suggests that the size of rental payments and proportion of spending on rice can help to identify the poor but remain more difficult to measure than assets and living environment. A second model that excludes the more difficult to measure consumption variables can be used to identify most of those initially defined as poor at the expense of some specificity (larger proportion of non-poor identified as poor).

There is a predominance of asset-based approaches to creating socio-economic groupings in data-sets such as DHS, Multi-Indicator Cluster Survey, Global Adult Tobacco Survey, STEPs NCD risk factor survey that are then used as the basis for policy targeting across sectors. The analysis here suggests that these approaches are limited and may be misleading in some contexts such as informal urban areas. Where asset measures are used alone, our quantitative and qualitative analysis has emphasised how poverty caused by high rents and loan repayments may be masked by the outward appearances of a well-built home. The vulnerability of those living in informal settlements to floods, fires and evictions and their transience of residence can play a complex role in distorting interpretation of assets. Understandably, households are reluctant to invest precious resources in assets that cannot easily be moved. The need to understand and respond to the issues of informality in the urban context is a theme that is gaining welcome exposure as approaches to monitoring the Sustainable Development Goals, particularly SDG 11, are developed [30]. Our study suggests that such efforts need to be reflected in the measurement of vulnerability to ensure that programmes can be reliably targeted at those in most need.

Migration status, particularly forced by economic circumstance, is shown to be associated with both level and types of poverty found in urban areas. Development theory suggests that populations move when there is an expected economic gain from the gap between incomes in urban area and actual income in original rural location [31, 32]. Theory predicts that while people will on average be better off, some will be substantially worse off with larger inequalities. Empirical studies, for example in Vietnam, suggest that while there is a long term gain from migration, those that moved are more likely to report lower job satisfaction and earnings than longer term migrants [33]. A study in Bangladesh suggested both larger inequalities and inferior health status amongst the urban migrant population [34]. Our study suggests that the effect on poverty of overall migrant status is small. However, individuals who recently migrated out of economic necessity are more likely to be both deprived and in consumption poverty than other groups. This is particularly the case with those that have recently migrated and suggests that targeting should particularly focus on this group.

Limitations on the findings and conclusions drawn arise from the nature of the data set used. The analysis in the paper is based on a snapshot survey of the urban population in the Kathmandu valley. However, the scope of the survey limits the extent to which country-wide conclusions can be drawn even for other urban populations. The one-off nature of the study, means that we are unable to fully explore the dynamics of poverty although the migration information allows some temporal tracking of the effects on poverty of time since migration. The qualitative data collected through participatory methods enabled an in-depth exploration of urban poverty from the perspective of urban communities. However, the qualitative data was collected in purposively sampled areas to explore issues in informal and poor neighbourhoods, so these were not co-terminus with the primary sampling units randomly selected for the household survey. This means that further analysis of issues in our survey sample areas of the issues raised could not be conducted. The qualitative analysis drove the choice of variables used in the construction of the urban poverty index. Since the instruments were implemented in parallel, not all of the community- identified variables were available although proxies were used in most cases. This weakness could be mitigated by ensuring that the qualitative analysis precedes the finalisation of the quantitative instrument.

## Conclusion

Money and capital measures of poverty assess distinct features of urban poverty. Ignoring one of the dimensions may ignore important aspects of vulnerability. A composite index of socioeconomic status can be estimated from the characteristics of households but some of the components related to acute poverty remain difficult to assess. A second version of the index that exclude the hard to measure consumption variables identifies most of those defined as by the UPI as poor but at the expense of some specificity. Those migrating out of economic necessity are particularly vulnerable compared to other migrants and the stable population. They are likely to lack immediate money resources but also have less physical, human and social capital to assist survival.

## Supporting information

S1 Annex Participatory methods and participant characteristics in the two sites. (DOCX)

S2 Annex Main features of poor, medium and better-off households from qualitative data and proxy quantitative indicators from household survey. (DOCX)
