# Supplementary material for: From Rags to Riches: Assessing poverty and vulnerability in urban Nepal
Source: PLoS One. 2020 Feb 5;15(2):e0226646. doi: 10.1371/journal.pone.0226646 (PMC7001899; doi:10.1371/journal.pone.0226646)
Supplement: S2 Annex — (DOCX) [file pone.0226646.s002.docx]

**S2 Annex. Main features of poor, medium and better-off households from qualitative data and proxy quantitative indicators from household survey.**
